# Responding to COVID-19: Implementing a Telemedicine Program at a Student-Run Free Clinic

**DOI:** 10.1089/tmr.2020.0037

**Published:** 2021-03-11

**Authors:** Elizabeth Cook, Bianca Arboleda, Heather Stewart, Eliza Nguyen, Alexander Shahin, Lucy Guerra, Eduardo Gonzalez

**Affiliations:** ^1^ University of South Florida Morsani College of Medicine, Tampa, Florida, USA.; ^2^ Division of General Internal Medicine, Department of Internal Medicine, University of South Florida Morsani College of Medicine, Tampa, Florida, USA.; ^3^ Department of Family Medicine, University of South Florida Morsani College of Medicine, Tampa, Florida, USA.

**Keywords:** student-run free clinic, telehealth, uninsured, Hispanic, COVID-19

## Abstract

**Introduction:**

Telemedicine has enabled access to care during the COVID-19 pandemic. This article describes the creation and implementation of a telemedicine clinic in a student-run free clinic (SRFC) serving uninsured patients in Tampa, FL.

**Methods:**

A new workflow was developed for a telemedicine clinic, including a screening algorithm to determine appropriateness for telemedicine appointments. Volunteer students and providers conducted patient remote visits that allowed students to have service-learning experiences. Analysis of patient visits between March 31, 2020, and July 23, 2020, was conducted. Study protocol was reviewed by the Institutional Review Board and an exemption was obtained.

**Results:**

Eighty-four visits were conducted for 58 unique patients. Seventy-two percent were female and 88% were of Hispanic or Latino origin. Forty-four students and 33 physicians volunteered. The majority of visits were general follow-ups (83%) followed by psychiatry (11%) and cardiology (6%).

**Conclusion:**

Telemedicine is a viable method of providing care for an at-risk uninsured population at an SRFC. It can also enhance service learning for medical student volunteers.

## Introduction

Telemedicine has been part of the medical landscape for many years.^1^ It has been shown to be a cost-effective and efficient means of delivering health care without compromising quality or patient satisfaction.^2–5^ Remote care has also been highlighted as a potential solution to decrease disparities among communities with limited access to care, including geriatric and rural populations.^6–10^ Before the COVID-19 pandemic, telemedicine, which includes both synchronous and asynchronous interactions, remote monitoring, wearable devices, and mobile applications, had been growing in popularity among patients and providers.^2,11^ However, the adoption of technology-enhanced care was slow, driven predominantly by technological innovations, health care worker shortages, and increasing patient demand for readily accessible health care services.^4,11,12^ Some specialties, such as psychiatry, cardiology, and radiology, were early adopters of this emerging care modality, a shift facilitated by Medicare and Medicaid reimbursement.^2,6,13^ In primary care, telemedicine has been implemented as a tool to meeting the needs of a growing population in the face of limited resources.^2,4–7^

The COVID-19 pandemic marked a dramatic shift in the practice of medicine, as providers across all specialties were required to find alternative means of delivering safe and effective care. This led to the rapid integration of telehealth into clinical practice to provide access to health care while reducing exposure to health care workers and patients alike. In the wake of COVID-19, many practices went from having no telehealth capability to having the majority of appointments through phone or video calling.^14–16^ This transition took place on a scale ranging from single clinics serving several hundred patients, to health care systems serving over a million people.^14,16^

The widespread adoption of telehealth across nearly all medical disciplines has prompted innovative solutions to existing barriers while throwing others into sharp relief. Some of the barriers encompass new organizational and technological factors including funding, developing new workflows, training staff, privacy concerns, software and hardware selection, and connectivity.^3,16–18^ Others are an extension of the widespread health care disparities within our medical system, including patient access to broadband internet, access to devices that support telehealth calls, low tech literacy, low health literacy, and limited English proficiency.^1,2,13–15,19^ As such, rural and ethnic minorities, patients of low socioeconomic status, and other vulnerable populations are the most likely to be affected by these barriers.^3,14,15,19^ In an analysis of their own care system's transition to telehealth, Nouri et al. found early signs of disparity in the use of telehealth in their older adult and non-English–speaking patient populations.^19^ Their experience highlights the care that must be taken to ensure that these populations are not left behind in the shift toward technology-enhanced health care.

Student-run free clinics (SRFCs) are one resource that may mitigate noted disparities in telemedicine. Owing to their flexibility of operation and their ability to provide low-cost care to underinsured or uninsured patients, SRFCs play an important role as a safety net provider within their communities.^20^ In a 2014 survey of Association of American Medical Colleges (AAMC) member institutions, >75% were found to have at least one associated SRFC, more than double the number that existed 9 years prior. SRFCs offer needed care in the areas of chronic disease management, specialty services, imaging, laboratories, pharmaceuticals, and interdisciplinary services.^21^

### Building Relationships and Initiatives Dedicated to Gaining Equality Healthcare Clinic

The Building Relationships and Initiatives Dedicated to Gaining Equality (BRIDGE) Healthcare Clinic is an SRFC that provides primary care to uninsured patients below the 200% poverty level in Hillsborough County, Tampa, FL. The clinic is led by physician, faculty, and student volunteers from various medical disciplines including medicine, physical therapy, pharmacy, public health, and social work. BRIDGE Healthcare Clinic is supported by the University of South Florida Morsani College of Medicine, generous community donors, and a multitude of dedicated volunteer staff.

### COVID-19 and BRIDGE

Historically, BRIDGE Healthcare Clinic has opened once weekly at the Morsani Center for Advanced Healthcare, an outpatient center located on the University of South Florida campus. In March 2020, a public health emergency was declared in Florida as part of the response to COVID-19.^22^ Along with AAMC restrictions on student participation in patient care, this resulted in the temporary closure of the clinic, and removal of medical student volunteers from patient-contact areas.^23^ Given these restrictions, alternative means of providing care to the BRIDGE patient population were required. This article describes the creation and implementation of a telehealth clinic in an established SRFC. Although much has been published over the past year regarding the shift to telehealth as a response to COVID-19, little has been written regarding the response to COVID-19 in free clinics serving at-risk populations. In this study, we address this gap in the literature and offer solutions to centers serving similar patient populations.

## Methods

In accordance with Centers for Disease Control and Prevention (CDC) guidelines to limit in-person interactions, BRIDGE Healthcare Clinic required patients to be seen through a virtual visit whenever possible.^24^ These virtual visits were offered at different days and times throughout the week as students, providers, and patients schedules allowed. We continued offering limited in-person visits for patients unable to be seen through telehealth during this time. Descriptions of our telehealth model of care and workflows are given hereunder. Our results represent findings from the study period March 31, 2020, to July 23, 2020. This study received an exemption from the internal review board.

### Telehealth platform

We used the HIPAA compliant telehealth platform, Doxy.me (Doxy.me Rochester, NY), to conduct virtual patient visits, as it was best suited to our needs and the needs of our patients. First, it requires minimal technological literacy to use, which was important for our patient population. Second, it was accessible through mobile phone, a requirement for the majority of our patients who do not have access to a computer. Third, Doxy.me allowed group calling, which was important for allowing live interpreters and multiple providers to join virtual visits. Fourth, Doxy.me offered substantial discount for free clinics.

### Telehealth training

Student directors of the BRIDGE Healthcare Clinic developed a medical student orientation guide that outlined the process of conducting virtual visits, including general workflow, proper attire, virtual care etiquette, and conducting a remote physical examination. Students were also instructed to complete the American College of Physicians online course titled, “Telemedicine: A practical guide for incorporation into your practice.”^25^

### Patient screening

**FIG. 1.**
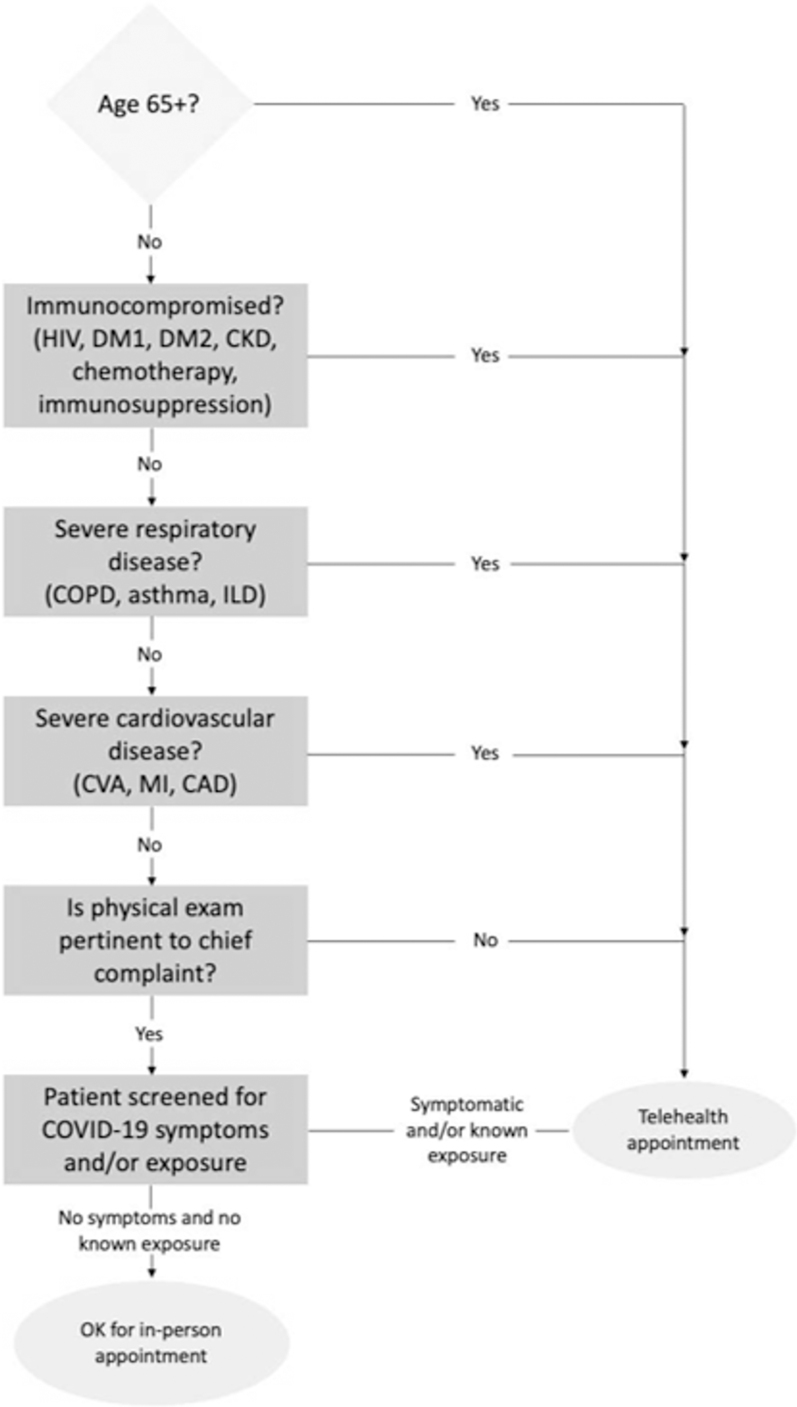
Screening algorithm for in-person versus telehealth appointments during COVID-19.

**FIG. 2.**
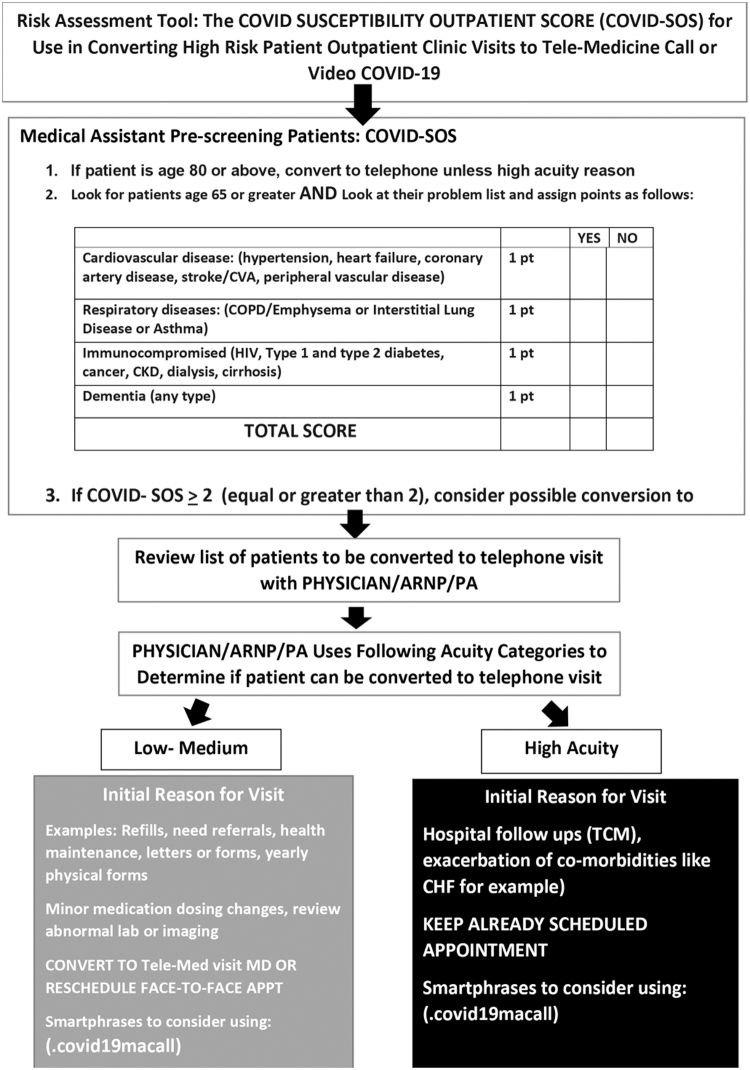
A COVID-19 risk assessment tool developed by Dr. Shivani Jani, Dr. Fancisco Alvarado, Dr. Fanco Garcia, Dr. Lucy Guerra, and Dr. Asa Oxner of the University of South Florida General Internal Medicine Team using the following references: Guan W. Clinical characteristics of coronavirus disease 2019 in China; Zhou F. Clinical course and risk factors for mortality of adult inpatients with COVID-19 Wuhan, China: a retrospective cohort study; and Ruan, Q. Clinical predictors on mortality due to COVID-19 based on analysis of data of 150 patients from Wuhan, China.

Before each clinic, patients were prescreened using a screening algorithm to determine whether in-person or telehealth visit was warranted (Fig. 1). This algorithm was derived from an evidence-based screening tool developed by providers in the department of internal medicine at the University of South Florida (Fig. 2), which used the most up-to-date scientific evidence to determine susceptibility to COVID-19. Patients at high risk of severe effects from COVID-19, those for whom physical examination was able to be performed through video, or those who only required counseling, such as explanation of laboratory or test results, were scheduled for a virtual visit. Patients at low risk of severe effects due to COVID-19, who also required an in-person physical examination for appropriate care, were permitted to schedule an in-person appointment.

### Patient triage and communication

**FIG. 3.**
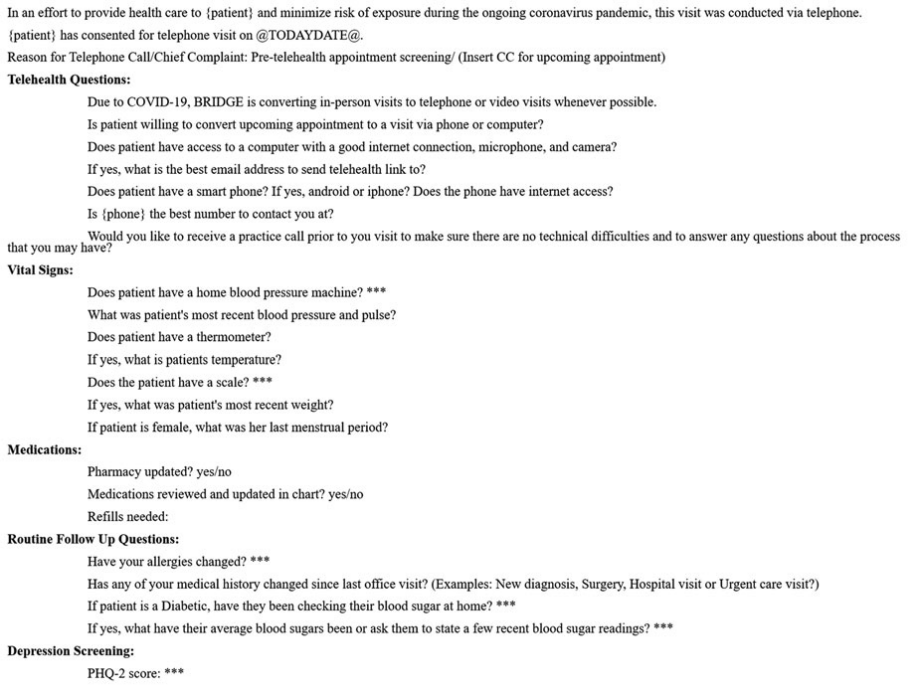
Telehealth triage note template used by clinic staff 1 week before the patient's telehealth appointment.

Within 1 week of the scheduled appointment, clinic staff called patients and administered a telehealth triage screening phone call. The purpose of this phone call was to assess the patients' current health status, including recent COVID-19 symptoms or exposure, current weight, blood pressure trends, menstrual history, and acute complaints. It was also used to confirm contact information and technological capabilities, for example, ownership of a video-enabled device and internet access. The telehealth triage template is shown in Figure 3.

One day before the scheduled appointment, a text message or email containing an appointment reminder and the Doxy.me patient link was sent through CareMessage, a secure text messaging service.

### Virtual clinic workflow

Patient care teams consisting of an upperclassmen medical student (third or fourth year), an interpreter, and a physician preceptor were arranged before each clinic. Each team was assigned up to two patients per clinic.

Immediately before clinic, a member of the clinic's staff conducted a virtual orientation session through Microsoft Teams (Microsoft Redmond, WA). This Microsoft Teams channel remained open for the duration of clinic in order for staff to provide assistance to the medical teams when needed.

The medical student began the patient encounter by logging into the Doxy.me platform. The encounter began once the student added the patient from the virtual waiting room to the private virtual clinic room. The medical student and interpreter completed the patient's history and performed a focused physical examination. After the initial portion of the encounter, the student added the physician preceptor to the virtual visit to finish the appointment. Upon completion of the encounter, students were required to complete the following tasks: fill out an online “patient checkout form,” fill out an online “patient tracker,” and complete the clinical note. The patient checkout form included information regarding orders, such as laboratories, prescriptions, imaging requests, referrals, and follow-up visits. In the patient tracker, the student logged which volunteer providers were involved in care, and the duration of each portion of the visit. A telemedicine summary was generated by staff members at the conclusion of the clinic to document all orders and referrals. The requests were reviewed by the office manager and faculty advisors for final approval.

### Volunteer staff roles

Volunteer staff roles were incorporated to improve clinic workflow and efficiency. Roles were divided between the staff coordinator (SC), patient coordinator (PC), and operations coordinator (OC), which were previously established roles for our in-person clinic. For our virtual clinic, the SC's responsibilities included notifying volunteers of their assigned patient care teams and recruiting volunteer medical students and physician preceptors. The role of the PC was to conduct the virtual orientation session and provide technical support to patients and providers during the encounters. Responsibilities of the OC included reviewing the postclinic checkout form and patient tracker, calling in prescription orders, entering laboratory orders, and creating the postclinic summary.

### Specialty and interdisciplinary care

Providers of specialty care continued regular patient visits through telehealth. In particular, we offered remote psychiatry and cardiology services. An attending physician conducted remote gynecological care with established patients; however, student volunteers were not included in these virtual visits. Our social work team also conducted remote patient visits. They used the same general workflow already highlighted with notable exceptions including not completing the checkout and patient tracker forms.

### Medical student education

With the transition of many clinical activities to telemedicine, the telemedicine clinic was integrated into the formalized learning experiences for the third-year primary care clerkship and some fourth-year family medicine electives. Students attended the virtual BRIDGE Healthcare Clinic as part of their clinical coursework where they were able to be supervised and evaluated by a physician preceptor.

## Results

**FIG. 4.**
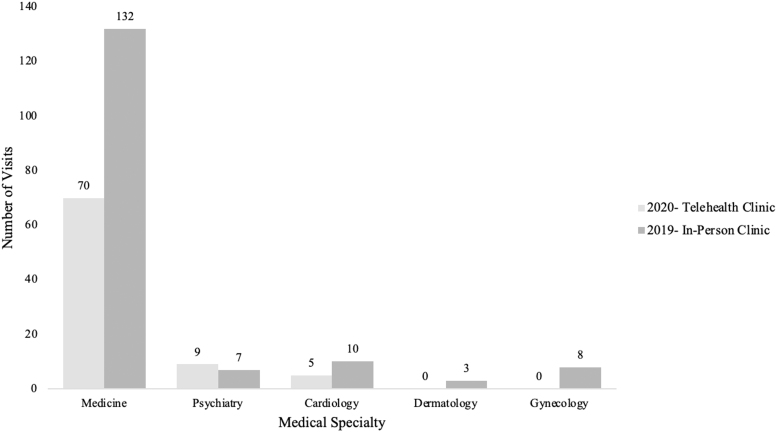
Total patient visits based on medical specialty for telehealth compared with in-person clinic held at the same time in 2019.

Eighty-four telemedicine visits were conducted with 54 unique patients during the study period. Eighty-three percent of visits were medicine follow-ups, whereas the remaining 11% and 6% were psychiatry and cardiology visits, respectively (Table 1). The majority of patients were Hispanic at 64%, which is significantly less than the 79% of patients who were Hispanic in the previous year, *z* = 5.62, *p* < 0.01. The telemedicine visits had a significantly higher percentage of patients' race not specified at 26% compared with 11%, *z* = 2.66, *p* > 0.01. The total number of patient visits per medical specialty for telehealth compared with the in-person clinic the same time period for the previous year is shown in Figure 4. Overall, there were significantly less patient visits for telehealth compared with the in-person clinic, χ^2^(4, *n* = 244) = 43.2, *p* < 0.01. For some specialties such as psychiatry, the number of visits increased for telehealth as compared with the in-person clinic during the same period the year prior. Eleven percent of visits were initial or follow-up encounters for COVID-19–positive patients. The telehealth clinics had a total of 44 student and 33 physician volunteers. Thirty-eight out of the 84 telehealth visits (45%) required an interpreter. Forty-four percent of visits were through the Doxy.me platform, whereas the remaining 56% were through phone call. Notably, there was an increase in the number of telemedicine visits conducted through Doxy.me rather than telephone in the month of July (71%) compared with the previous months. Telemedicine visits generated 21 referrals, with ophthalmology being the most common (19%, *n* = 4). Laboratories were ordered in 61 out of the 84 visits (73%), whereas imaging was ordered in 19 out of the 84 visits (23%). On average, 1.7 medications were ordered per visit, with the most common being for hypertension (12%), diabetes (12%), and hyperlipidemia (11%).

**Table 1.** Patient Demographics of Telehealth Clinic Compared with In-Person Clinic

Parameter	Telehealth clinic (March 31–July 23, 2020)	In-person clinic (March 31–July 23, 2019)
	All patients (*N* = 58)	All patients (*N* = 130)
Age, years, mean ± SD	49 ± 10	48 ± 12
(range)	(29–77)	(21–89)
Male	16 (28%)	35 (27%)
Female	42 (72%)	95 (73%)
Race		
Hispanic	37 (64%)	103 (79%)^a^
White	2 (3%)	7 (5%)
Black or African American	2 (3%)	5 (4%)
Multiracial	2 (3%)	1 (1%)
Did not specify	15 (26%)^a^	14 (11%)

^a^ Significant difference noted when comparing clinics.

SD, standard deviation.

## Discussion

The development of a telemedicine clinic in response to COVID-19 served three principal purposes: it provided continuity of care for uninsured patients in our community, allowed for the provision of safe care to COVID-19–positive patients, and created opportunities for medical education. Our experience demonstrates the utility of telemedicine in addressing the health disparities of vulnerable populations served by an SRFC.

### Continuity of care

A notable benefit of this telemedicine initiative is that it allowed our uninsured patients to continue receiving care for both acute and chronic medical problems. It also offered greater flexibility in caring for patients with barriers to traditional care, such as longer or shifted work hours, limited transportation, and significant childcare needs due to the pandemic. Our experience supports existing literature showing telemedicine to be a quality means of providing medical care.^2,4,7,10,11,13^

Patient volume for our virtual general medicine appointments was ∼50% of our typical in-person clinic. The observed decrease in patient volume for this clinic was multifactorial. As this was BRIDGE Healthcare Clinic's first experience with telemedicine, the decision was made to cap the total number of patients during each clinic, to maintain high quality of care and ensure that an adequate number of staff members were available to address technical difficulties. Another limiting factor in the volume of virtual visits in our clinic was a noted decline in the number of licensed physicians available to volunteer. Pandemic-related constraints, including mandatory quarantines and increased physician demand both in inpatient and outpatient settings, limited our volunteer workforce, even on a virtual basis. We were able to mitigate this to some extent by scheduling additional patient visits outside of our regular Tuesday evening clinic based on provider and patient availability, which was made possible by the inherent flexibility of telemedicine. In addition, our triage process required rescheduling annual physical and new patient visits thereby decreasing total patient volume.

The psychiatry specialty clinic maintained a virtual patient volume that was approximately the same as in-person clinic. This is unsurprising, given that telemedicine interventions have been well documented within the field of psychiatry. Because physical examination findings pertinent to psychiatric care can be easily gathered by video, the field is well suited to virtual care.^2,16,26^ Our cardiology providers participated in virtual patient visits, but at a decreased volume comparable with that observed for our general medicine clinic, and for similar reasons.

In-house gynecology and dermatology have historically been part of BRIDGE Healthcare Clinic's specialty services; however, neither been offered through telemedicine to date. Despite the fact that gynecology is well established at BRIDGE, nonurgent gynecological care, including well-woman examinations, was deferred due to the frequent need to perform a pelvic examination. By contrast, the in-house dermatology clinic at BRIDGE is relatively new and, therefore, relied on providers less familiar with BRIDGE operations and its patient population. Dermatology visits were deferred until the reopening of our in-person clinic.

### COVID response

The telemedicine clinic played a critical role in our ability to respond appropriately to the COVID-19 pandemic. Specifically, it served as an early point of contact for patients who had possible exposures or COVID-like symptoms. As a result, we were able to see eight patients with confirmed or suspected COVID-19 infection. Our triage and screening methods also facilitated the timely referral of COVID-19–positive patients to COVID-19–specific clinics to ensure that they received appropriate follow-up. These referrals also enabled patients to receive necessary resources that were beyond the scope of BRIDGE Healthcare Clinic.

### Medical education

Providing quality service-learning experiences for medical students at all levels is integral to the mission of BRIDGE Healthcare Clinic. Owing to ongoing concerns for student safety, it was impossible to continue offering traditional in-person service learning; however, students were able to participate virtually. Utilizing a novel workflow, upper level medical students were able to participate in patient encounters. Owing to the AAMC mandated removal of medical students from clinical settings, these students would have otherwise had minimal patient contact during this time.^23^ Notably, participation of first- and second-year medical students in virtual care has not been possible thus far. This is due to the increasing complexity of virtual visits and concerns for decreasing video and audio quality with additional participants. Furthermore, the educational benefit of telemedicine for less-experienced students has not been determined.

### Telemedicine and health disparities

Given the potential barriers to telemedicine among our patient population, and the acute need to address the impact of COVID-19 among vulnerable patients in our community, we prioritized creating a telemedicine system that allowed us to provide quality services while also mitigating the exacerbation of existing disparities in care. Demographically, our telemedicine patients were not different in terms of age and ethnicity than patients seen in our in-person clinic. This is notable for several reasons. BRIDGE Healthcare Clinic patients are largely of Hispanic or Latino ethnicity; all are uninsured, with annual incomes at least 200% below the federal poverty line. Literature suggests that under-resourced populations experience greater barriers to health care, including access to telemedicine. Furthermore, recent studies have documented disparities in the incidence of COVID-19, as well as COVID-19–related morbidity and mortality among ethnic minority communities.^19,27,28^ Likewise, the pandemic has disproportionately affected uninsured patients due to increased exposure, including greater reliance on public transportation and decreased ability to quarantine.^29^

SRFCs are uniquely positioned to address health care inequities, as they provide regular care to populations most in need. Telemedicine represents a powerful tool toward achieving that goal. Although literature suggests that racial and ethnic minority and low socioeconomic status populations experience barriers to technology-enhanced care, we prioritized the development of features in our telemedicine program that mitigated potential barriers.^19^ For example, low tech literacy and low rates of smartphone ownership among uninsured individuals have been noted as a significant obstacle to participation in technology-enhanced care.^29,30^ These factors greatly impact the ability to access telemedicine appointments.^19^ Given the evidenced-based benefits of video-enabled telemedicine as opposed to audio alone, we prioritized developing a system in which our patients could access their appointments with relative ease.^31^ The triage phone call was critical to addressing this issue, as it was an opportunity for staff to address questions about the virtual encounter and determined patients' technological capabilities. As a result, 44% of patient encounters utilized Doxy.me, whereas only 56% were by audio only. High rates of smartphone ownership among our patient population also contributed to our success with a video platform. We addressed potential difficulties with connecting to telemedicine appointments due to low tech literacy in multiple ways. We selected Doxy.me as our telehealth platform, which does not require a software download or a user account. Doxy.me is user friendly, as patients access their appointment by clicking on a single link. In addition, clinic staff members were on call to assist patients in connecting to their telehealth appointments when necessary.

Furthermore, limited English proficiency is a well-documented barrier to utilization of telehealth services.^14,19,29^ As the BRIDGE Healthcare Clinic patient population is primarily Spanish speaking, we have traditionally relied on in-person interpreters to assist with translation needs. To address this issue remotely, we integrated interpreters into the telemedicine clinic workflow when needed. Their immediate availability remotely, either by Doxy.me or by phone, played a critical role in the success of the clinic.

Finally, literature on barriers to in-person care for low-income and minority populations suggests that time and scheduling constraints are among the most significant obstacles.^32,33^ We note anecdotally that patients express high levels of satisfaction with telemedicine due to the flexibility of appointments and our ability to provide care outside of our regular Tuesday evening clinic. This feature may be a facilitator to accessing care for underserved populations.

### Limitations

At this point, we have not been able to fully assess our patient comprehension in telemedicine visits in comparison with in-person visits. This study is also limited by the telemedicine clinic being created with an already established SRFC. It does not address the difficulties of creating a stand-alone telemedicine clinic.

### Future directions

Although the benefits of large-scale telemedicine interventions are well established, future studies should quantify patient satisfaction with telemedicine and evaluate their ability to understand their care, relative to in-person visits. There is also opportunity to assess the completion of referrals, laboratory orders, and imaging ordered through telehealth compared with the completion of those ordered in-person. This would allow us to assess our ability to mitigate language and technological barriers to care with our current workflow. In addition, prior quality improvement projects have demonstrated that the BRIDGE Healthcare Clinic model has helped patients achieve clinically significant improvements in health outcomes, such as blood pressure control.^34^ It would be valuable to determine whether this benefit still exists for patients managed through telemedicine. Although some of our patients already possess the means to monitor their blood pressures at home, an area of improvement relating to this effort would be in providing all BRIDGE patients with automated blood pressure cuffs for both screening and monitoring purposes. Ideally, an initiative to provide this resource for our patients would be expanded to include home blood glucose monitoring or pulse oximetry where appropriate. Furthermore, given the mission of BRIDGE Healthcare Clinic to provide service-learning opportunities to students of all levels, methods to incorporate junior medical students (first- and second-year students) into telehealth will be explored. Likewise, we plan to integrate interdisciplinary care such as public health, pharmacy, and physical therapy into the telehealth format, to provide the holistic care offered in our in-person clinics. Finally, as in-person operations resume, the feasibility of making telehealth a permanent component of weekly clinic will also be addressed.

## Conclusion

The creation and implementation of a telemedicine clinic allowed BRIDGE Healthcare Clinic to continue providing care to low-income and racial and ethnic minority patients in a context wherein in-person care was not possible. This resulted in the ability to triage and provide close follow-up of COVID-19–positive patients while minimizing exposure to volunteers and patients. At the same time, we created a novel workflow tailored to address the educational goals of an SRFC. Our experience demonstrates that telemedicine is a viable opportunity to address health care disparities in vulnerable populations.

## Abbreviations Used

AAMC: Association of American Medical Colleges
BRIDGE: Building Relationships and Initiatives Dedicated to Gaining Equality
OC: operations coordinator
PC: patient coordinator
SC: staff coordinator
SD: standard deviation
SRFC: student-run free clinic
